# Associations of Physical Activity and Heart Rate Variability from a Two-Week ECG Monitor with Cognitive Function and Dementia: The ARIC Neurocognitive Study

**DOI:** 10.3390/s24134060

**Published:** 2024-06-21

**Authors:** Francesca R. Marino, Hau-Tieng Wu, Lacey Etzkorn, Mary R. Rooney, Elsayed Z. Soliman, Jennifer A. Deal, Ciprian Crainiceanu, Adam P. Spira, Amal A. Wanigatunga, Jennifer A. Schrack, Lin Yee Chen

**Affiliations:** 1 Department of Epidemiology, Johns Hopkins Bloomberg School of Public Health, Baltimore, MD 21205, USA; 2 Center on Aging and Health, Johns Hopkins Bloomberg School of Public Health, Baltimore, MD 21205, USA; 3 Courant Institute of Mathematical Sciences, New York University, New York, NY 10012, USA; 4 Welch Center for Prevention, Epidemiologic, and Clinical Research, Johns Hopkins Bloomberg School of Public Health, Baltimore, MD 21205, USA; 5 Department of Cardiology, Wake Forest University School of Medicine, Winston-Salem, NC 27109, USA; 6 Cochlear Center for Hearing and Public Health, Johns Hopkins Bloomberg School of Public Health, Baltimore, MD 21205, USA; 7 Department of Biostatistics, Johns Hopkins Bloomberg School of Public Health, Baltimore, MD 21205, USA; 8 Department of Mental Health, Johns Hopkins Bloomberg School of Public Health, Baltimore, MD 21205, USA; 9 Department of Psychiatry and Behavioral Sciences, Johns Hopkins School of Medicine, Baltimore, MD 20205, USA; 10 Lillehei Heart Institute, Department of Medicine, University of Minnesota Medical School, Minneapolis, MN 55455, USA

**Keywords:** physical activity, heart rate variability, dementia, accelerometry, ECG, digital health, remote monitoring

## Abstract

Low physical activity (PA) measured by accelerometers and low heart rate variability (HRV) measured from short-term ECG recordings are associated with worse cognitive function. Wearable long-term ECG monitors are now widely used, and some devices also include an accelerometer. The objective of this study was to evaluate whether PA or HRV measured from long-term ECG monitors was associated with cognitive function among older adults. A total of 1590 ARIC participants had free-living PA and HRV measured over 14 days using the Zio^®^ XT Patch [aged 72–94 years, 58% female, 32% Black]. Cognitive function was measured by cognitive factor scores and adjudicated dementia or mild cognitive impairment (MCI) status. Adjusted linear or multinomial regression models examined whether higher PA or higher HRV was cross-sectionally associated with higher factor scores or lower odds of MCI/dementia. Each 1-unit increase in the total amount of PA was associated with higher global cognition (β = 0.30, 95% CI: 0.16–0.44) and executive function scores (β = 0.38, 95% CI: 0.22–0.53) and lower odds of MCI (OR = 0.38, 95% CI: 0.22–0.67) or dementia (OR = 0.25, 95% CI: 0.08–0.74). HRV (i.e., SDNN and rMSSD) was not associated with cognitive function. More research is needed to define the role of wearable ECG monitors as a tool for digital phenotyping of dementia.

## 1. Introduction

Physical activity (PA) is a known modifiable risk factor for cognitive impairment and dementia [1,2,3]. There is a growing interest in objectively measuring PA using accelerometers. These devices are small, non-invasive, and continuously capture free-living movement over multiple days [4]. This method has important advantages for measuring PA in older adult populations who typically spend most of their time being sedentary or performing lighter intensity PA [4]. There are many different brands of accelerometers currently used in research [4]. Yet, there are barriers to integrating these devices into clinical environments [5]. Recent work from our group found that accelerometry data measured by the Zio^®^ XT Patch, a commonly used ambulatory electrocardiogram (ECG) monitor capable of continuous (i.e., uninterrupted) wear for up to 14 days, was highly correlated with ActiGraph wrist accelerometry data [6]. To determine the potential utility of wearable ECG monitors with embedded accelerometers, it is important to evaluate associations between accelerometer-measured PA and important clinical outcomes, such as cognitive impairment. However, whether PA measured from these ECG monitors is related to cognitive function has not yet been established.

Heart rate variability (HRV) is defined as variation in successive heartbeats and lower HRV is associated with poor health outcomes [7,8]. Previous studies among adults without cognitive impairment have shown that lower HRV is associated with poorer global cognition [9,10], memory [9,11], language [9], attention [9], executive function [9,12], visuospatial performance [9], and processing speed [9]. Lower HRV is also related to lower cognitive test performance among those with cognitive impairment [13] and neurodegenerative diseases [14]. This prior work is based on HRV measures from ECG recordings taken over several minutes in clinical settings or 24 h recordings taken in a free-living environment. Longer ECG recordings might be more accurate in representing an individual’s HRV, particularly in response to the environment [7,15]. To date, no studies have evaluated associations between HRV from long-term ECG recordings and cognitive function.

Thus, we aimed to address these gaps by leveraging data from Zio^®^ XT Patch ECG (iRhythm Technologies, San Francisco, CA, USA). and accelerometry data measured over 14 days in a free-living environment. First, we evaluated the cross-sectional association of total PA with cognitive function among older adults. Second, we examined the cross-sectional association between long-term HRV and cognitive function. We hypothesized that lower total PA and lower HRV are associated with lower cognitive test performance and higher odds of dementia compared to higher PA or HRV, respectively.

## 2. Methods

### 2.1. Study Population

**Figure 1:**
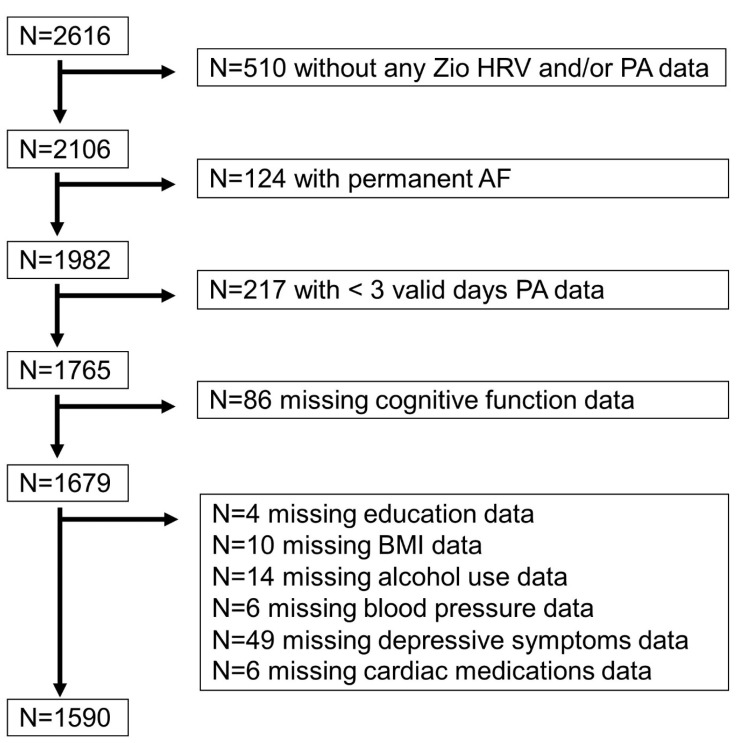
Flowchart of sample selection. Note: PA = physical activity, AF = atrial fibrillation, BMI = body mass index.

The Atherosclerosis Risk in Communities (ARIC) study is a prospective cohort study of 15,792 community-dwelling, mostly Black and White, adults aged 45–64 years at enrollment. Participants were enrolled from Forsyth County, North Carolina, Jackson, Mississippi, Minneapolis, Minnesota, or Washington County, Maryland, between 1987 and 1989 [16]. This analysis included participants without permanent atrial fibrillation who attended visit 6 in 2016–2017 and wore the Zio^®^ XT Patch (iRhythm Technologies, San Francisco, CA, USA). Participants missing cognitive tests, dementia status, or covariate data were excluded (Figure 1). All study protocols were approved by the institutional review board at each study site, and all participants provided informed consent.

### 2.2. Heart Rate Variability

HRV measures were derived from continuous ECG raw data over 14 days from the Zio^®^ XT Patch. The patch was placed on the participant’s chest and worn continuously until the end of data collection. After data collection, the participant mailed the patch to iRhythm Technologies Inc for data processing. The primary HRV measures of interest included two HRV indices in the time domain: (1) the standard deviation of normal-to-normal RR intervals (SDNN) and (2) the root mean squared successive difference in normal-to-normal RR intervals (rMSSD). Lower SDNN and rMSSD are associated with poor health outcomes [7,8]. RR intervals are the time between successive normal heartbeats, SDNN represents the standard deviation between heartbeats, and rMSSD describes beat-to-beat variance [7]. SDNN and rMSSD were log-transformed due to non-normal distributions and then analyzed continuously. Additional details of HRV data processing can be found in the supplementary materials (heart rate variability data processing).

### 2.3. Physical Activity

First, accelerometry raw data from the Zio^®^ XT Patch tri-axial accelerometer were summarized into minute-level mean amplitude deviation (MAD), a measure of movement intensity with gravitational units [17]. MAD is strongly correlated with activity counts [18], heart rate [17], and PA intensity [17]. A recent study has also validated MAD from the Zio^®^ XT Patch against total activity counts measured using the ActiGraph wrist accelerometer [6]. Second, minute-level MAD measurements were screened for periods of wear and non-wear based on the simultaneously measured ECG data. Third, days with more than 144 (10%) non-wear minutes were excluded and participants with fewer than three valid days were excluded from further analysis. Finally, minute-level data were summarized into participant-level total mean amplitude deviation (TMAD), which is the daily total MAD averaged over all valid days. Higher TMAD can therefore be interpreted as higher levels of PA [17,18]. TMAD was natural-log-transformed due to non-normal distributions and then analyzed continuously (LTMAD). Processing of minute-level activity data was performed using the arctools R package [19].

### 2.4. Cognitive Test Factor Scores

Cognitive test performance was defined by factor scores for global cognition, executive function, memory, and language at visit 6. Participants completed a neuropsychological test battery with tests administered by trained psychometrists [20]. Executive function tests included the Trail Making Test parts A and B [21] and Digit Symbol Substitution Test [22]. Memory tests included delayed word recall and logical memory from the Wechsler Memory Scale—Revised [23] and incidental learning from the Wechsler Adult Intelligence Scale III [24]. Language tests included semantic and phonemic fluency [25] and Boston Naming tests [26]. From these individual cognitive tests, cognitive factor scores for global cognition, executive function, memory, and language were derived using confirmatory factor analysis [20]. Confirmatory factor analysis is a latent variable approach that uses all available cognitive test data to generate factor scores with a common scale while accounting for the common covariation among cognitive tests [20]. Missing cognitive test data were addressed by using maximum likelihood with robust standard errors [20]. Advantages of confirmatory factor analysis include: (1) use of all available test data, (2) allowing individual tests to have unequal weights, and (3) accounting for measurement error of individual tests [20]. The full details of the cognitive factor score derivation in ARIC are described elsewhere [20].

### 2.5. Mild Cognitive Impairment or Dementia Status

Participants were classified as having dementia, mild cognitive impairment (MCI), or unimpaired cognition at visit 6. Classifications were algorithmically based on criteria from the National Institute on Aging—Alzheimer’s Association workgroups [27,28] and from the 5th Edition Diagnostic and Statistical Manual of Mental Disorders [29]. This algorithm used information from the Mini Mental State Exam [30], Clinical Dementia Rating Scale Sum of Boxes [31], cognitive tests, and Functional Activities Questionnaire [32] to define participants as having highly likely or probable unimpaired cognition, MCI, dementia, or an indeterminate status [33].

Participants were defined as having MCI following at least one cognitive test < 1.5 standard deviations below the mean, a cognitive test decline below the 10th or 20th percentiles for one or two tests, respectively, a Clinical Dementia Rating Scale Sum of Boxes score > 0.5 but ≤3, and a Functional Activities Questionnaire score ≤ 5 [33]. Participants were defined as having dementia following at least two cognitive tests < 1.5 standard deviations below the mean, a cognitive test decline below the 10th or 20th percentiles for one or two tests, respectively, a Clinical Dementia Rating Scale Sum of Boxes score > 3, and a Functional Activities Questionnaire score > 5 [33]. Mini Mental State Examination scores < 21 for White participants or < 19 for Black participants also defined a dementia classification [33]. Participants were classified as cognitively unimpaired (i.e., normal cognition) if they did not meet the criteria for MCI or dementia [33]. Data were reviewed by a panel of clinicians for participants classified as having MCI or dementia that was highly likely or probable or who had an uncertain status. The full details of MCI and dementia classifications in ARIC are described elsewhere [33].

### 2.6. Other Covariates

The following covariates were measured at visit 1: age (years), sex (male, female), race/center, and education (grade school, high school without degree, high school graduate, vocational school, at least some college, or graduate/professional school). Covariates measured at visit 6 included: smoking status (current, former, never), alcohol status (current, former, never), body mass index (BMI; kilograms/meters^2^), systolic and diastolic blood pressure (mmHg), history of diabetes, history of heart failure, depressive symptoms (Center for Epidemiologic Studies Depression Scale score, which ranges from 0–60 with higher scores indicating higher depressive symptoms) [34], and cardiac medications (beta blocker, digoxin, calcium channel blocker, or anti-arrhythmic). Each medication was individually included as a covariate.

### 2.7. Statistical Methods

To provide basic summaries of the analytic sample, participants were categorized into PA and HRV tertiles as there are no accepted clinical cut-offs for these measures. Variables were summarized as tertile-specific means and standard deviations or as counts and proportions, and differences between tertiles were tested using F-tests or chi-squared tests for continuous or categorical variables, respectively.

The objective of this study was to evaluate associations between PA or HRV measured from a long-term ECG monitor with cognitive test performance and dementia status. To accomplish this objective, separate linear regression models estimated the mean cross-sectional difference in global cognition, memory, executive function, or language factor scores per 1-unit increment in LTMAD, log SDNN, or log rMSSD. Separate multinomial logistic regression models estimated the odds of MCI or dementia versus unimpaired cognition per 1-unit increment in LTMAD, log SDNN, or log rMSSD. To visually evaluate differences in PA by dementia status, diurnal curves of MAD over 24 h were plotted by dementia status. The final adjusted models included age, sex, race/center, education, smoking, alcohol, systolic and diastolic blood pressure, BMI, diabetes, heart failure, depressive symptoms, and each cardiac medication.

The models described above were fit with additional sample exclusions or adjustments in sensitivity analyses. First, LTMAD, log SDNN, and log rMSSD were included in the same adjusted models to determine whether there was an independent effect of PA above and beyond HRV or vice versa. Second, individuals with a history of stroke and/or non-permanent atrial fibrillation (i.e., intermittent atrial fibrillation) or who were taking beta blockers or calcium channel blockers were excluded, as these conditions and medications might confound the associations between PA or HRV with cognitive function. Third, those with MCI or dementia were excluded from the cognitive test score models to evaluate whether results were driven by participants with cognitive impairment. All analyses were conducted using Stata statistical software version 17 (StataCorp, College Station, TX, USA), and *p*-values < 0.05 were considered to be statistically significant.

## 3. Results

### 3.1. Sample Characteristics

Of the N = 2616 participants who attended ARIC visit 6 and wore the Zio^®^ XT Patch, 1590 participants were included in the analysis (Figure 1). The overall sample characteristics of these 1590 participants are described in Table 1 and Table 2. Participants were, on average, aged 78.8 (range: 72–94) years, 58% were female, and 32% were Black. Participants exhibiting higher PA were younger, less likely to be Black, and more likely to be male, current drinkers, and former smokers compared to those with lower PA. This group was also associated with higher education attainments, lower body mass index and systolic blood pressure, and lower prevalence of diabetes, heart failure, and depressive symptoms (Table 1). Participants with higher HRV were less likely to be Black, more likely to be male, had lower BMI, and lower prevalence of diabetes than those with lower HRV (Table 2).

TMAD was weakly correlated with SDNN and with rMSSD (Pearson’s correlation = 0.22 or −0.08, respectively). The two HRV measures (i.e., SDNN and rMSSD) were moderately correlated (Pearson’s correlation = 0.48) (Figure S1).

### 3.2. Physical Activity and Cognitive Function

The first objective of this study was to evaluate associations between PA measured from a long-term ECG monitor with cognitive test performance. In fully adjusted models, each 1-unit increment in LTMAD was significantly associated with a 0.30 increase in global cognition (95% CI: 0.16, 0.44) and a 0.38 increase in executive function factor scores (95% CI: 0.22, 0.53). There were no associations with memory or language factor scores (Table 3).

**Figure 2:**
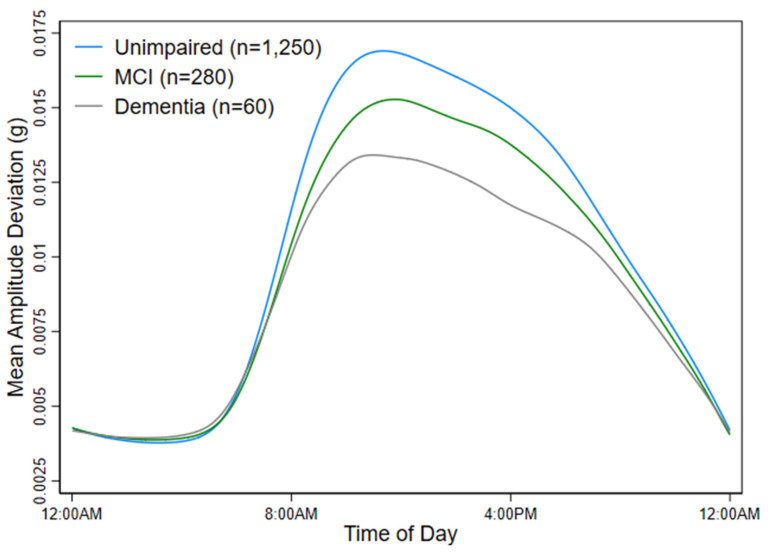
Diurnal patterns of MAD by dementia, mild cognitive impairment, or cognitively unimpaired status.

An additional objective of this study was to evaluate associations between PA and the odds of MCI/dementia. When looking at the diurnal curves of PA in Figure 2, participants without cognitive impairment (blue line) had the highest peak in activity. This figure displays differences in patterns of PA over 24 h by dementia status. This group also exhibited higher daytime activity than those with MCI (green line) or dementia (gray line). Participants with MCI and dementia had blunted diurnal patterns of activity, with those with dementia exhibiting the greatest attenuation in activity (Figure 2). In fully adjusted models, each 1-unit increment in LTMAD was associated with a 62% decrease in the odds of MCI (OR = 0.38, 95% CI: 0.22, 0.67) or a 75% decrease in the odds of dementia (OR = 0.25, 95% CI: 0.08, 0.74) versus unimpaired cognition (Table 4).

### 3.3. Heart Rate Variability and Cognitive Function

The second objective of this study was to evaluate associations between HRV measured from a long-term ECG monitor and cognitive test performance and MCI/dementia. There were no significant associations between log SDNN or log rMSSD and cognitive function. SDNN and rMSSD were not significantly associated with cognitive test performance (Table 3) nor with MCI or dementia (Table 4).

### 3.4. Independent Effects of Physical Activity and Heart Rate Variability Measures

The first sensitivity analysis aimed to evaluate whether PA or HRV was independently associated with cognitive function. When including LTMAD, log SDNN, and log rMSSD together in the same model, associations for higher LTMAD with higher global cognition and executive function factor scores and lower odds of MCI or dementia remained significant. Further, a 1-unit increment in LTMAD was associated with a 0.15 increase in memory factor scores (95% CI: 0.04, 0.33). Associations with log SDNN or log rMSSD and cognitive factor scores or dementia status remained non-significant (Tables S1 and S2).

### 3.5. Exclusion of Stroke, Intermittent Atrial Fibrillation, Beta Blockers, and Calcium Channel Blockers

The second sensitivity analysis further controlled for possible confounding effects associated with chronic conditions or disease status. When excluding N = 170 participants with any history of stroke or intermittent atrial fibrillation and N = 675 taking either beta blockers or calcium channel blockers, associations between LTMAD, on one hand, and global cognition, executive function factor scores, dementia, on the other, remained significant (Tables S3 and S4). Further, each 1-unit increment in log SDNN was associated with a 0.59 increase in executive function factor scores (95% CI: 0.12, 1.07) (Table S3).

### 3.6. Sensitivity Analyses Restricted by Cognitive Status

The final sensitivity analysis evaluated whether associations were driven by participants with cognitive impairment. When excluding N = 340 participants with MCI or dementia, associations between LTMAD and executive function factor scores remained robust, but global cognition findings were non-significant (*p* = 0.09). Associations between log SDNN or log rMSSD and cognitive factor scores remained non-significant (Table S5).

## 4. Discussion

In a community-based sample of older adults, higher levels of free-living PA derived from an accelerometer embedded in a wearable ECG monitor worn by participants for two weeks was cross-sectionally associated with a 0.30 increase in global cognition factor scores, a 0.38 increase in executive function factor scores, and a 62% or 75% decrease in the odds of MCI or dementia. By contrast, time domain measures of HRV derived from the 2-week ECG raw data were not associated with cognitive performance and MCI/dementia. These findings raise the possibility of using accelerometers embedded within wearable ECG monitors as a tool to obtain digital phenotyping of dementia. These findings also challenge previously reported associations between lower HRV and poorer cognitive function based on short-term heart rate data.

Higher PA levels measured using the Zio^®^ XT Patch were cross-sectionally associated with a 0.30–0.38 increase in executive function and global cognition factor scores, as well as a 62% decrease in the odds of MCI or a 75% decrease in the odds of dementia. These findings were largely robust across multiple sensitivity analyses. This is consistent with prior research documenting an association between higher PA and better cognitive performance [35] and lower risk of dementia [1,2,3], as well as research reporting that individuals living with MCI/dementia exhibit differences in objectively measured PA patterns [36]. Potential mechanisms explaining these associations include higher PA leading to increased brain neurotrophic factors [35,37] and cerebral blood flow [35,37] or decreased inflammation [35], stress overactivity [35], and cardiovascular risk factors [2,38]. In addition, it is important to acknowledge that emerging evidence suggests that associations between cognition and PA might be bidirectional [39]. However, we are unable to establish claims about temporality from this cross-sectional study. While this work supports the potential utility of accelerometers embedded in long-term wearable ECG monitors for the routine measurement of free-living PA, future longitudinal studies are needed to determine whether these data can be used to predict cognitive decline or dementia.

In our analyses, HRV was not related to cognitive test performance or odds of MCI/dementia. Previous works using short-term measurements of HRV suggest that higher values of certain indices are associated with better cognition, but other studies report null results [9,12,40]. The non-significant findings in our study might be due to the measurement of free-living HRV over a longer ECG monitoring period of 14 days which more accurately represents an individual’s HRV compared with shorter ECG recordings of several minutes to 24 h [15]. Further, our study focused on only two time-domain measures that are relatively constant over time [7,41]. Frequency-domain measures (i.e., high frequency, low frequency, etc.) which divide total HRV into certain frequency categories and represent different physiological systems [9] might be more strongly related to cognitive function. Future studies are needed to evaluate whether other patterns of long-term HRV are related to cognitive function.

In sensitivity analyses excluding those with a history of stroke, atrial fibrillation, beta blockers, or calcium channel blockers, we found that higher HRV, specifically SDNN, was associated with a 0.59 increase in executive function factor scores. These results align with studies finding associations between higher HRV and better executive function [9,11,12,40,42]. HRV is influenced by both components of the autonomic nervous system [7] and represents an individual’s ability to adapt to changes in their environment (i.e., faster heart rate with stressor) [9]. Certain diseases and medications can impact HRV, including stroke [43], atrial fibrillation [44], and cardiac medications [45]. Having a history of stroke or atrial fibrillation is also associated with a cognitive decline and risk of dementia [46]. As such, these factors might have hidden (i.e., confounded) the association between HRV and cognitive function when analyzing the whole sample. The mechanisms linking HRV with cognitive function should be evaluated in future work, while also considering potential differences in associations caused by certain cardiovascular diseases or medications.

This study suggests the possible utility of accelerometers embedded in wearable ECG monitors as a tool to assess cognitive function and provide digital phenotyping of dementia. This has important public health implications, as many wearable ECG monitors are already commonly used in the clinical setting. Further, the measure of PA used in this study was a simple summary of the total amount of activity, adding to the feasibility of using these data. We have previously shown that this measure of total amount of PA is highly correlated with total activity counts from the ActiGraph accelerometer [6]. Although the summary measures of HRV were not related to cognitive function in this study, it is possible that more detailed metrics of long-term HRV are associated with cognitive function. Specifically, it might be necessary to examine HRV by time of day. Higher daytime HRV is associated with positive health outcomes [7,8], whereas higher overnight HRV is linked with negative health outcomes [47]. It might also be important to consider whether bouts of high HRV but low PA, or vice versa, are informative of cognitive function. This could include combining minute-level ECG and accelerometry data to generate time-varying profiles of PA and HRV. Beyond exploring additional HRV measures, replication of these findings in longitudinal cohorts with more ethnically diverse samples is needed, as the findings from this cross-sectional study do not inform on whether PA measured from this ECG monitor can predict cognitive decline or dementia.

### Limitations and Strengths

It is important to consider these results in the context of the study’s limitations. First, we are not able to establish temporality from these cross-sectional analyses. There is a possibility for reverse causation, where those with lower cognitive function might be less active and therefore have lower HRV. Second, we do not have information available on the context of activities that the participants were performing, information which would help interpret the PA and HRV data. Third, there is possible selection bias, as participants must have had both Zio^®^ XT Patch and cognitive function data to be included in this analysis. If individuals with dementia or low cognitive test performance and worse PA or HRV did not wear the ECG device, then we would expect this bias to be conservative (i.e., attenuated findings).

This study also has strengths. First, the ARIC study is a well-characterized cohort with a large sample of White and Black older adults. Second, ARIC conducts a comprehensive neuropsychological test battery and rigorous ascertainment of dementia, allowing us to examine associations across multiple cognitive domains and by dementia status. Third, we evaluated PA and HRV measured by a common clinical device in the free-living environment over 14 days.

## 5. Conclusions

Higher free-living PA measured over two weeks by a wearable continuous ECG monitor is positively associated with cognitive performance and MCI/dementia. By contrast, long-term time-domain measures of HRV are not associated with cognitive performance or MCI/dementia. Our findings suggest the potential of accelerometers embedded in wearable continuous ECG monitors as a promising tool for digital phenotyping of dementia. Future research is needed to evaluate more detailed digital measures of PA and HRV and to evaluate whether these indices can predict cognitive decline or dementia.

## Figures and Tables

**Table 1:** Sample characteristics overall and by tertiles (low, medium, high) of LTMAD.

	Overall (N = 1590)	Low LTMAD (N = 530)	Medium LTMAD (N = 530)	High LTMAD (N = 530)
Age (years), mean (SD)	78.8 (4.5)	80.5 (5.0)	78.8 (4.3)	77.1 (3.4)
Female, %	58	65	59	48
Black, %	32	42	29	26
Education, %				
Grade school	3	5	2	3
Some high school	7	9	6	7
High school graduate	28	30	27	26
Vocational school	10	9	11	10
At least some college	34	30	38	33
Grad/prof school	18	17	16	22
BMI (kg/m^2^), mean (SD)	28.4 (5.4)	29.9 (6.2)	28.5 (5.2)	26.8 (4.0)
Alcohol use, %				
Current	52	42	53	60
Former	28	31	29	23
Ever	20	27	18	16
Smoking, %				
Current	8	9	8	6
Former	50	45	52	52
Never	43	46	41	42
BP (mm Hg), mean (SD)				
Systolic	134.6 (18.7)	135.5 (19.6)	135.4 (18.6)	133.0 (17.9)
Diastolic	67.7 (10.4)	67.2 (10.3)	67.7 (10.7)	68.3 (10.3)
Diabetes, %	30	38	30	23
Heart Failure, %	6	12	5	2
Cardiac medications, %	50	59	50	42
CES-D, mean (SD)	2.5 (2.8)	3.2 (3.1)	2.5 (2.8)	2.0 (2.2)
TMAD, mean (SD)	16.6 (4.7)	12.0 (1.7)	16.0 (1.2)	21.9 (3.6)
SDNN (ms), mean (SD)	125.3 (39.9)	119.0 (42.3)	123.3 (37.2)	133.7 (38.5)
rMSSD (ms), mean (SD)	47.2 (46.9)	52.1 (52.4)	43.8 (38.7)	45.6 (48.2)
Cognitive status, %				
Unimpaired	79	72	80	84
MCI	17	22	17	14
Dementia	4	6	3	2

LTMAD = natural log of total mean amplitude deviation; SDNN = standard deviation of normal-to-normal RR intervals; rMSSD = root mean squared successive deviation of normal-to-normal RR intervals; BMI = body mass index; BP = blood pressure; CES-D = Center for Epidemiologic Studies Depression Scale; MCI = mild cognitive impairment.

**Table 2:** Sample characteristics overall and by tertiles (low, medium, high) of log SDNN.

	Overall (N = 1590)	Low SDNN (N = 530)	Medium SDNN (N = 530)	High SDNN (N = 530)
Age (years), mean (SD)	78.8 (4.5)	78.8 (4.5)	78.7 (4.4)	78.8 (4.4)
Female, %	58	69	60	44
Black, %	32	36	34	26
Education, %				
Grade school	3	3	4	3
Some high school	7	8	8	6
High school graduate	28	29	27	26
Vocational school	10	11	10	9
At least some college	34	32	34	35
Grad/prof school	18	16	17	21
BMI (kg/m^2^), mean (SD)	28.4 (5.4)	29.2 (6.0)	28.2 (5.1)	27.8 (4.8)
Alcohol use, %				
Current	52	49	50	56
Former	28	28	31	25
Ever	20	23	19	19
Smoking, %				
Current	8	10	6	7
Former	50	48	51	49
Never	43	43	43	43
BP (mm Hg), mean (SD)				
Systolic	134.6 (18.7)	135.1 (19.4)	135.2 (18.5)	133.7 (18.3)
Diastolic	67.7 (10.4)	68.8 (10.9)	67.3 (9.9)	67.0 (10.4)
Diabetes, %	30	37	30	24
Heart Failure, %	6	9	5	4
Cardiac medications, %	50	59	49	44
CES-D, mean (SD)	2.5 (2.8)	2.8 (3.0)	2.4 (2.7)	2.4 (2.6)
TMAD, mean (SD)	16.6 (4.7)	15.1 (3.9)	16.9 (4.5)	17.9 (5.3)
SDNN (ms), mean (SD)	125.3 (39.9)	89.0 (15.9)	119.8 (9.9)	167.1 (36.3)
rMSSD (ms), mean (SD)	47.2 (46.9)	32.2 (21.3)	41.6 (28.0)	67.8 (68.5)
Cognitive status, %				
Unimpaired	79	79	79	79
MCI	17	18	17	18
Dementia	4	4	4	4

TMAD = total mean amplitude deviation; SDNN = standard deviation of normal-to-normal RR intervals; rMSSD = root mean squared successive deviation of normal-to-normal RR intervals; BMI = body mass index; BP = blood pressure; CES-D = Center for Epidemiologic Studies Depression Scale; MCI = mild cognitive impairment.

**Table 3:** Cross-sectional differences in cognitive test scores by LTMAD, log SDNN, or log rMSSD over 24 h.

	Global Cognition β (95% CI)	Executive Function β (95% CI)	Memory β (95% CI)	Language β (95% CI)
LTMAD	0.30 (0.16, 0.44)	0.38 (0.22, 0.53)	0.14 (−0.04, 0.32)	0.15 (−0.02, 0.32)
Log SDNN	0.03 (−0.24, 0.30)	0.19 (−0.10, 0.48)	−0.21 (−0.56, 0.13)	−0.15 (−0.47, 0.17)
Log rMSSD	−0.12 (−0.2, 0.010)	−0.08 (−0.2, 0.06)	−0.16 (−0.3, 0.004)	−0.11 (−0.26, 0.05)

Linear regression model adjusted for: age, sex, race/center, education, smoking, drinking, systolic and diastolic blood pressure, body mass index, diabetes, heart failure, depressive symptoms, and cardiac medications; N = 1590.

**Table 4:** Cross-sectional odds ratio of MCI or dementia compared to unimpaired cognition by 1-unit increment in LTMAD, log SDNN, or log rMSSD over 24 h.

	Unimpaired (N = 1250)	Mild Cognitive Impairment (N = 280) OR (95% CI)	Dementia (N = 60) OR (95% CI)
LTMAD	REF	0.38 (0.22, 0.67) ^a^	0.25 (0.08, 0.74)
Log SDNN	REF	0.84 (0.30, 2.36)	3.39 (0.40, 28.37)
Log rMSSD	REF	1.40 (0.87, 2.27)	1.24 (0.46, 3.35)

Multinomial logistic regression model adjusted for: age, sex, race/center, education, smoking, drinking, systolic and diastolic blood pressure, body mass index, diabetes, heart failure, depressive symptoms, and cardiac medications; N = 1590. a. 1-unit increase in LTMAD (i.e., more physical activity) is associated with a 62% decrease in the odds of MCI relative to unimpaired cognition.

## Data Availability

Researchers with an approved manuscript proposal form may request data from the Atherosclerosis Risk in Communities Coordinating Center.

## Supplementary Materials

The following supporting information can be downloaded at: https://www.mdpi.com/article/10.3390/s24134060/s1, Supplementary Materials include supplementary methods, figures, and tables. Supplementary methods: Heart rate variability data processing. Supplementary Figure S1: Correlations among log TMAD (LTMAD), log SDNN, and log rMSSD. Supplementary Table S1: Cross-sectional differences in cognitive factor scores by LTMAD, log SDNN, or log rMSSD adjusted for LTMAD, log SDNN, and log rMSSD together. Supplementary Table S2: Cross-sectional odds of MCI or dementia compared to unimpaired cognition by LTMAD, log SDNN, or log rMSSD adjusted for LTMAD, log SDNN, and log rMSSD together. Supplementary Table S3: Cross-sectional differences in cognitive factor scores by LTMAD, log SDNN, or log rMSSD excluding participants with a history of stroke, any atrial fibrillation, and use of beta blockers or calcium channel blockers. Supplementary Table S4: Cross-sectional odds of MCI or dementia compared to unimpaired cognition by LTMAD, log SDNN, or log rMSSD excluding participants with a history of stroke, any atrial fibrillation, and use of beta blockers or calcium channel blockers. Supplementary Table S5: Cross-sectional differences in cognitive factor scores by LTMAD, log SDNN, or log rMSSD among cognitively unimpaired participants. References [48,49,50,51,52,53] have been cited in the Supplementary Materials.
