# Supplementary material for: Associations of Physical Activity and Heart Rate Variability from a Two-Week ECG Monitor with Cognitive Function and Dementia: The ARIC Neurocognitive Study
Source: Sensors (Basel). 2024 Jun 21;24(13):4060. doi: 10.3390/s24134060 (PMC11244549; doi:10.3390/s24134060)
Supplement: Supplementary file 1 [file sensors-24-04060-s001.zip › sensors-3022251-supplementary.pdf]

sensors MDPI

SUPPLEMENTARY MATERIALS

Supplemental Methods: Heart Rate Variability Data Processing

To obtain SDNN and rMSSD, the following steps were carried out. The power line interference was filtered using a notch filter at 60 Hz. The baseline wandering was filtered using a highpass filter with the cutoff at 5/8Hz, and a lowpass filter with the cutoff 75Hz was applied to suppress noise. The ECG signal was divided into 5 second nonoverlapping epochs. All epochs with the 99.9% percentile less than 30 mV were flagged as artifact. R peaks were detected by a variation of the Pan-Tompkins algorithm [1]. When two consecutive peaks were detected with the period shorter than the refractory period 250 ms, the peak with the sharpest slope was retained. Suppose the polarity was corrected if an rS pattern was seen. The bSQI [2] with another R peak detection algorithms by the de-shape algorithm with the peak tracking algorithm [3] was applied to each epoch not flagged artifacts with an output F1 between 0 and 1. All epochs with F1 less than 0.9 were masked as low quality.

To reduce the impact of artifacts incurred by the spurious or missing R peak detection, we applied the following global edit. If a suspected RR interval was longer than 3-seconds, it was possible that one or more R peaks were missed, and we took the median of the closest 50 RR intervals shorter than 3 seconds to estimate the underlying RR interval, and divided the suspected interval into k segments, where k was the rounding integer of the division of the suspected RR interval and the median. To reduce the impact of arrhythmic beats, particularly the premature ventricular contractions (PVC) and premature atrial contractions (PAC), we applied a quantile-based edit [4] of the RRI time series. First, if an RR interval was dramatically greater than the 90% quantile or less than the 10% quantile of the previous 25 normal RRIs associated with normal cardiac cycles, it was labeled as a suspected RR interval. Second, if a suspected RRI was shorter than the 10% quantile of previous 25 normal RRIs, this suspected RR interval was removed. Third, if a suspected RR interval was longer than the 90% quantile of the previous 25 normal RRIs, the interval was divided into k segments, where k is the rounding integer of the division of the suspected RR interval and the median of previous 25 normal RRIs. To reduce the impact of atrial fibrillation, we considered the low-complex RR-interval based atrial fibrillation detection algorithm [5] and marked those segments with atrial fibrillation episodes.

With the corrected RR interval time series, we calculated the traditional time-domain HRV indices, including the SDNN and rMSSD in the following way [6]. RRIs within the marked artifact epochs were removed, and SDNN and rMSSD were evaluated in the remaining RRIs with the unit ms.

References

1. Malik, J.; Soliman, E.Z.; Wu, H.T. An adaptive QRS detection algorithm for ultra-long-term ECG recordings. *J. Electrocardiol.* **2020**, *60*, 165–171. https://doi.org/10.1016/j.jelectrocard.2020.02.016.
2. Johnson, A.E.W.; Behar, J.; Andreotti, F.; Clifford, G.D.; Oster, J. Multimodal heart beat detection using signal quality indices. *Physiol. Meas.* **2015**, *36*, 1665–1677. https://doi.org/10.1088/0967-3334/36/8/1665.
3. Su, L.; Wu, H.T. Extract Fetal ECG from Single-Lead Abdominal ECG by De-Shape Short Time Fourier Transform and Nonlocal Median. *Front. Appl. Math. Stat.* **2017**, 3. https://doi.org/10.3389/fams.2017.00002.
4. Xu, X.; Schuckers, S.; CHIME Study Group. Collaborative Home Infant Monitoring Evaluation. Automatic detection of artifacts in heart period data. *J. Electrocardiol.* **2001**, *34*, 205–210. https://doi.org/10.1054/jelc.2001.28876.
5. Petrėnas, A.; Marozas, V.; Sörnmo, L. Low-complexity detection of atrial fibrillation in continuous long-term monitoring. *Comput. Biol. Med.* **2015**, *65*, 184–191. https://doi.org/10.1016/j.compbiomed.2015.01.019.
6. The Task Force of the European Society of Cardiology, The North American Society of Pacing. Heart rate variability: Standards of measurement, physiological interpretation and clinical use. Task Force of the European Society of Cardiology and the North American Society of Pacing and Electrophysiology. *Circulation* **1996**, *93*, 1043–1065.

sensors MDPI

Figure S1. Correlations between log TMAD (LTMAD), log SDNN, and log rMSSD.

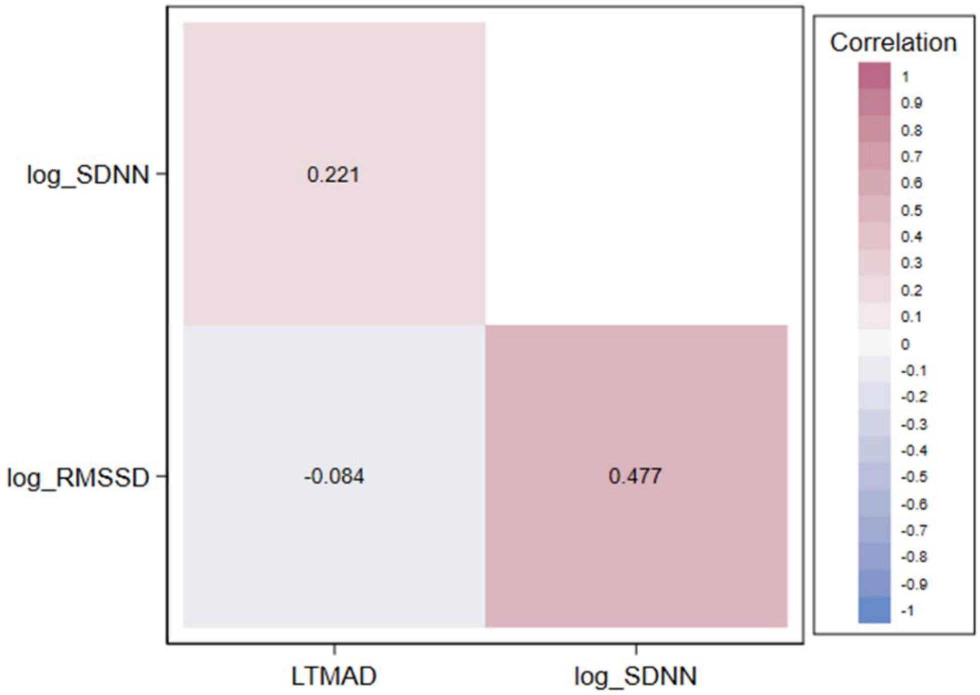

Table S1. Cross-Sectional Differences in Cognitive Factor Scores by LTMAD, log SDNN, or log rMSSD Adjusting for LTMAD, log SDNN, and log rMSSD Together.

	Global Cognition β (95% CI)	Executive Function β (95% CI)	Memory β (95% CI)	Language β (95% CI)
LTMAD [a]	0.29 (0.14, 0.43)	0.36 (0.20, 0.51)	0.15 (0.04, 0.33)	0.16 (-0.01, 0.33)
Log SDNN [b]	0.09 (-0.23, 0.41)	0.23 (-0.11, 0.57)	-0.12 (-0.53, 0.29)	-0.11 (-0.49, 0.27)
Log rMSSD [b]	-0.14 (-0.28, 0.01)	-0.13 (-0.29, 0.03)	-0.13 (-0.32, 0.06)	-0.08 (-0.26, 0.10)

Linear regression model adjusted for: age, sex, race/center, education, smoking, drinking, systolic and diastolic blood pressure, body mass index, diabetes, heart failure, depressive symptoms, and cardiac medications; N=1590.

a. Model additionally adjusted for log SDNN and log rMSSD.

b. Model additionally adjusted for LTMAD.

Table S2. Cross-Sectional Odds of MCI or Dementia Compared to Unimpaired Cognition by LTMAD, log SDNN, or log rMSSD Adjusting for LTMAD, log SDNN, and log rMSSD Together.

	Unimpaired	Mild Cognitive Impairment OR (95% CI)	Dementia OR (95% CI)
LTMAD [a]	REF	0.40 (0.22, 0.70)	0.21 (0.07, 0.63)
Log SDNN [b]	REF	0.69 (0.20, 2.34)	8.56 (0.60, 121.59)
Log rMSSD [b]	REF	1.52 (0.86, 2.70)	0.68 (0.20, 2.31)

Multinomial logistic regression model adjusted for: age, sex, race/center, education, smoking, drinking, systolic and diastolic blood pressure, body mass index, diabetes, heart failure, depressive symptoms, and cardiac medications; N=1590 (overall).

a. Model additionally adjusted for log SDNN and log rMSSD.

b. Model additionally adjusted for LTMAD.

sensors

MDPI

Table S3. Cross-Sectional Differences in Cognitive Factor Scores by LTMAD, log SDNN, or log rMSSD Excluding Participants with History of Stroke, Any Atrial Fibrillation, Beta-Blockers, or Calcium Channel Blockers.

	Global Cognition β (95% CI)	Executive Function β (95% CI)	Memory β (95% CI)	Language β (95% CI)
LTMAD	0.21 (0.006, 0.42)	0.37 (0.15, 0.59)	0.06 (-0.20, 0.33)	0.10 (-0.15, 0.35)
Log SDNN	0.37 (-0.07, 0.81)	0.59 (0.12, 1.07)	-0.03 (-0.59, 0.53)	-0.12 (-0.65, 0.40)
Log rMSSD	-0.11 (-0.32, 0.10)	-0.07 (-0.30, 0.15)	-0.11 (-0.38, 0.15)	-0.17 (-0.42, 0.08)

Linear regression model adjusted for: age, sex, race/center, education, smoking, drinking, systolic and diastolic blood pressure, body mass index, diabetes, heart failure, depressive symptoms, and cardiac medications (excluding beta blockers or calcium channel blockers); N=745.

Table S4. Cross-Sectional Odds of MCI or Dementia Compared to Unimpaired Cognition by LTMAD, log SDNN, or log rMSSD Excluding Participants with History of Stroke, Any Atrial Fibrillation, Beta-Blockers, or Calcium Channel Blockers.

	Unimpaired	Mild Cognitive Impairment OR (95% CI)	Dementia OR (95% CI)
LTMSD	REF	0.56 (0.24, 1.30)	0.12 (0.02, 0.72)
Log SDNN	REF	0.35 (0.06, 2.04)	2.99 (0.08, 117.6)
Log rMSSD	REF	1.10 (0.47, 2.57)	0.88 (0.15, 5.15)

Multinomial logistic regression model adjusted for: age, sex, race/center, education, smoking, drinking, systolic and diastolic blood pressure, body mass index, diabetes, heart failure, depressive symptoms, and cardiac medications (excluding beta blockers or calcium channel blockers); N=745.

Table S5. Cross-Sectional Differences in Cognitive Factor Scores by LTMAD, log SDNN, or log rMSSD Among Cognitively Unimpaired Participants.

	Global Cognition β (95% CI)	Executive Function β (95% CI)	Memory β (95% CI)	Language β (95% CI)
LTMAD	0.12 (-0.02, 0.26)	0.24 (0.08, 0.40)	-0.006 (-0.2, 0.17)	-0.01 (-0.19, 0.17)
Log SDNN	0.03 (-0.24, 0.30)	0.21 (-0.09, 0.51)	-0.12 (-0.47, 0.23)	-0.13 (-0.48, 0.21)
Log rMSSD	-0.04 (-0.16, 0.09)	0.02 (-0.12, 0.17)	-0.10 (-0.26, 0.06)	-0.08 (-0.24, 0.08)

Linear regression model adjusted for: age, sex, race/center, education, smoking, drinking, systolic and diastolic blood pressure, body mass index, diabetes, heart failure, depressive symptoms, and cardiac medications; N=1590.
